# Monotropein attenuates apoptosis and pyroptosis in chondrocytes and alleviates osteoarthritis progression in mice

**DOI:** 10.1186/s13020-023-00748-2

**Published:** 2023-04-19

**Authors:** Zhen Li, Zhenyue Chen, Jiayi Chen, Zhutong Liu, Zehui Li, He Sun, Xiaochao Wang, Jinqiang Wei, Xuewei Cao, Decai Zheng

**Affiliations:** 1 The Second Clinical College of Guangzhou, University of Chinese Medicine, The Second Affiliated Hospital of Guangzhou University of Chinese Medicine, Guangdong Provincial Hospital of Chinese Medicine, Guangzhou, 510120 Guangdong China; 2 The First Clinical College of Guangzhou, University of Chinese Medicine, Guangzhou, 510405 Guangdong China; 3 Zhongshan Hospital of Traditional Chinese Medicine Affiliated to Guangzhou University of Traditional Chinese Medicine, Zhongshan, 528401 Guangdong China; 4 Department of Orthopaedic Surgery, Guangdong Provincial Hospital of Chinese Medicine, 111 Dade Road, Yuexiu District, Guangzhou, 510120 Guangdong China; 5 Department of Rehabilitation, Guangdong Provincial Hospital of Chinese Medicine, 261 Datong Road, Yuexiu District, Guangzhou, 510105 Guangdong China

**Keywords:** Monotropein, Osteoarthritis, Cartilage matrix degradation, Apoptosis, Pyroptosis

## Abstract

**Background:**

Osteoarthritis (OA) is a chronic degenerative joint disease characterized by loss of joint function, which seriously reduces the quality of life of the elderly and imposes a heavy socioeconomic burden worldwide. Monotropein (MON), the main active ingredient of *Morinda officinalis* F.C. How, has exhibited therapeutic effects in different disease models. However, its potential effects on chondrocytes in an arthritic model remain unclear. This study aimed to evaluate the effects of MON in chondrocytes and a mouse model of OA, and explore the potential mechanisms.

**Materials and methods:**

Murine primary chondrocytes were pretreated with 10 ng/ml interleukin (IL)-1β for 24 h to establish an in vitro model of OA, and then treated with different concentrations of MON (0, 25, 50 and 100 μM) for 24 h. The proliferation of the chondrocytes was assayed using ethynyl-deoxyuridine (EdU) staining. Immunofluorescence staining, western blotting and TUNEL staining were performed to assess the effects of MON on cartilage matrix degradation, apoptosis and pyroptosis. The mouse model of OA was constructed by surgical destabilization of the medial meniscus (DMM), and the animals were randomly divided into the sham-operated, OA and OA + MON groups. Following OA induction, the mice were given intraarticular injection of 100 μM MON or equal volume of normal saline twice a week for 8 weeks. The effects of MON on cartilage matrix degradation, apoptosis and pyroptosis were assessed as indicated.

**Results:**

MON significantly accelerated the proliferation of chondrocytes, and inhibited cartilage matrix degradation, apoptosis and pyroptosis in the IL-1β-stimulated cells by blocking the nuclear factor-kappa B (NF-κB) signaling pathway. In the mouse model as well, MON treatment alleviated OA progression and promoted cartilage repair by inhibiting cartilage matrix degradation, and chondrocyte apoptosis and pyroptosis through the inactivation of the NF-κB signaling pathway. Furthermore, the MON-treated arthritic mice exhibited better articular tissue morphology and lower OARSI scores.

**Conclusions:**

MON alleviated OA progression by inhibiting cartilage matrix degradation, and the apoptosis and pyroptosis of chondrocytes via NF-κB pathway inactivation, and is a promising alternative for the treatment of OA.

## Introduction

Osteoarthritis (OA) is a chronic degenerative joint disease with pathological features including articular cartilage degeneration, synovial inflammation, and secondary bone hyperplasia [2, 9]. It is characterized by a prolonged disease course, high morbidity and disability rates, and joint pain, dysfunction and deformity, which severely affects quality of life and imposes a heavy socioeconomic burden worldwide [12, 27]. OA is currently managed by basic treatment, pharmacological intervention, restorative treatments and reconstruction [19, 36], depending on the degree of arthritic progression [17]. However, these therapies only mitigate the symptoms, and cannot prevent or reverse the disease course [28]. Therefore, novel treatment strategies are need to inhibit the development and progression of OA. 

Monotropein (MON), a natural iridoid glycoside compound, is the main active ingredient of *Morinda officinalis* F. C. How, which is widely used for treating OA [47]. The plant name has been checked with http://www.worldfloraonline.org/ (2022. 11. 28). Pharmacological studies have shown that MON exerts anti-inflammatory, antioxidant, anti-apoptotic and autophagic effects. It alleviated inflammation, oxidative stress and apoptosis in senescent endothelial cells and mice with acute kidney injury by inhibiting the nuclear factor-kappa B (NF-κB) signaling pathway [15, 45]. In addition, Chen et al. reported that MON alleviated secondary liver injury in a mouse model of chronic colitis by inactivating the NF-κB pathway and NLR Family Pyrin Domain Containing 3 (NLRP3) inflammasome [4]. Furthermore, MON significantly attenuated H_2_O_2_-induced oxidative stress in osteoblasts by enhancing autophagy [31], and protected endothelial progenitor cells against apoptosis and autophagy [33]. 

Although MON has shown therapeutic effects in different diseases models, its possible effects on chondrocytes in an arthritic model remain unclear. Therefore, the aim of this study was to evaluate the therapeutic potential of MON in in vitro and in vivo models of OA, and explore the potential mechanisms.

## Materials and methods

### Primary culture of chondrocytes

Primary chondrocytes were extracted from neonate C57BL/6 mice as previously described [41, 42]. Briefly, the cartilage tissues were isolated from the knee joints under sterile conditions by removing the bone and connective tissues, washed thrice with PBS, and cut into small pieces. The minced cartilage tissues were then homogenized with 0. 25% trypsin (Gibco) for 30 min at 37 °C. After removing the supernatant, the tissues were rinsed with PBS and subsequently digested with 0. 2% collagenase type II (Sigma, Beijing, China) for 12 h at 37 °C on a shaker. The macerated tissue suspensions were passed through a nylon mesh to remove the residue, and then clarified by centrifuging at 1000 rpm for 5 min. The primary chondrocytes were resuspended in Dulbecco's Modified Eagle Medium (DMEM, Gibco) containing 10% fetal bovine serum (FBS; Gibco), 1% penicillin and streptomycin (Gibco), and seeded into tissue culture flasks. The cells were cultured at 37 °C under 5% CO_2_ and saturated humidity, and the medium was changed every 2 days. The second and third passages of chondrocytes were used for in vitro experiments at 80% confluency. 

### Cell viability assay

MON (≥ 98% purity) was purchased from Sigma-Aldrich Chemical Corporation (St Louis, MO, USA) and dissolved in 0. 9% normal saline. Cell viability was determined using the Cell Counting Kit-8 (CCK-8, KeyGEN, China) [23]. Briefly, primary chondrocytes were seeded into 96-well plates at the density of 5000 cells per well and incubated with different concentrations of MON (0–1000 μM) for 24 h. According to previous studies on drug concentration and concentration screening in this experiment, the optimal experimental concentration was confirmed [33, 34, 47]. In another experiment, the cells were first stimulated with 10 ng/ml IL-1β for 24 h, and then treated with MON (0, 25, 50 and 100 μM) for 24 and 48 h. At the indicated time points, 10 µl CCK-8 solution was added to the respective wells, and the cells were incubated for 2 h. The absorbance at 450 nm (OD450) was measured using a multifunctional microplate reader (Bio-Rad).

### Ethynyl-deoxyuridine (EdU) staining

Primary chondrocytes were seeded in 96-well plates, pre-treated with 10 ng/ml IL-1β for 24 h, and then incubated with MON (0, 25, 50 and 100 μM) for 24 h. Cell proliferation was assayed using BeyoClick™ EdU-488 Cell Proliferation Kit (Beyotime, Beijing, China) [38, 40]. The EdU-labeled cells were observed under a fluorescence microscope (Olympus IX73), and the proportion of proliferating cells were calculated using Image J.

### Establishment of OA model and treatment

Sixty-three 10-weeks-old male specific pathogen-free (SPF) C57BL/6 mice (20–25 g) were supplied by the Experimental Animal Center of Guangzhou University of Chinese Medicine. Fifteen mice were used in the preliminary test and forty-eight were used in the formal test. All animal experiments were conducted in accordance with the internationally accepted principles for laboratory animal use and care as found in the European Community guidelines (EEC Directive of 1986; 86/609/EEC). The OA model was constructed by surgical destabilization of the medial meniscus (DMM) as previously described [3]. Briefly, after the mice were anesthetized, their right knee was depilated and a 0. 5 cm long incision was made along the medial capsule of the right knee. The joint was then exposed and holding the patella firmly, the tissues surrounding the medial meniscus were bluntly dissected. The medial meniscus ligament was then dissected and displaced medially, thereby destabilizing the tissue. After suturing and sterilizing the wounds, the mice were placed under an electric blanket till they regained consciousness. 

The animals were randomly divided into the sham-operated, OA and OA + MON groups. Consistent with in vitro experiments, three concentration doses (25, 50 and 100 μM) were used to perform the animal pretest. According to the pre-experimental results, we found that the concentration of 100 μM MON showed significant statistical difference, so we used the single-dose concentration to illustrate the effect of MON. The mice in the OA + MON group were given intra-articular injections of 100 μM MON twice a week for 8 weeks after OA induction, and 10μL of the solution was slowly injected into right knees. Animals in the sham-operated and OA groups received equal volume of normal saline. After 8 weeks of MON treatment, all mice were sacrificed and the right knee joint was excised for further evaluation. 

### Histological assessment

After eight weeks of MON treatment, the animals were euthanized and the right knee tissue was fixed with 4% PFA and then decalcified with ethylenediaminetetraacetic acid (EDTA). After decalcification, the tissues were dehydrated through an ethanol gradient (70%, 80%, 95–100%), cleared with xylene, and embedded in paraffin. The wax blocks were sliced into 5 µm-thick sections that were baked overnight in an oven at 37 °C. The sections were dewaxed and stained 0. 2% Safranin O solution for 15 min, rinsed with distilled water, counterstained with 0. 2% Fast Green solution for 5 min (Sigma-Aldrich, USA), and sealed. The structural changes in the cartilage tissues were evaluated in a blinded manner according to the Osteoarthritis Research Society International (OARSI) scoring system [6]. Hematoxylin–eosin (HE) staining was performed as per standard protocols, and the inflammation around the lesions was scored. The slides were observed using the Olympus IX73 microscope. 

### Western blotting

The cartilage tissues were sonicated in the radio immunoprecipitation assay (RIPA) lysis buffer (Gibco, Grand Island, NY, USA), and centrifuged twice at 12000 g for 10 min at 4 °C. The protein concentration in the supernatants were measured using a bicinchoninic acid (BCA) kit (Bio-Rad Laboratories, CA, USA). Twenty micrograms protein per sample was diluted in the loading buffer and denatured at 99 °C for 10 min. The protein samples were separated by 10% SDS-PAGE, and then transferred to polyvinylidene difluoride (PVDF) membranes. After blocking with 5% skimmed milk, the blots were incubated overnight with primary antibodies targeting matrix metallopeptidase 3 (MMP3, Proteintech, 66338-1-lg), MMP13 (Proteintech, 18165-1-AP), collagen II (Abcam, ab34712), SOX9 (Proteintech, 67439-1-lg), Bcl-2 (CST, 15071), Bax (CST, 41162), cleaved caspase-3(CST, 9661), NLRP3(Affinity, DF7438), apoptosis-associated speck-like protein containing a CARD(ASC, CST, 67824), cleaved caspase-1 (CST, 89332), gasdermin D (GSDMD, Affinity, AF4012), phosphorylated IκBα (p-IκBα, Affinity, AF2002), IκBα(Affinity, AF5002), p-p65(CST, 3033), p65 (CST, 8242) and GAPDH (CST, 5174) at 4 °C. All primary antibodies were used at 1:1000 dilution. The membranes were incubated with the secondary antibody (Beyotime, 1:1000, A0208 anti-rabbit or A0216 anti-mouse) for 90 min at room temperature (RT). The positive bands were visualized using a ChemiDoc™ MP Imaging System (Bio-Rad), and the relative intensities of each band were determined using Image J (National Institutes of Health, Bethesda, MD). 

### Immunofluorescence staining

Immunofluorescence was performed as per standard protocols. The suitably treated chondrocytes were fixed with 4% PFA for 30 min and permeabilized in 0. 3% Triton X-100 at RT for 30 min. Tissue sections were treated with xylene and alcohol gradient, and heated in citric acid for antigen retrieval. After incubating with 10% serum for 1 h at RT to block non-specific binding, the cells or tissue sections were incubated overnight with primary antibodies against collagen II (1:200, Abcam, ab34712), MMP13 (1:200, Proteintech, 18165-1-AP), NLRP3 (1:200, Affinity, DF7438), GSDMD (1:100, Affinity, AF4012), p-IκBα (1:200, Affinity, AF2002) and p-p65 (1:1000, CST, 3033) at 4 °C. The following day, the cells or tissue sections were incubated with fluorochrome-conjugated rabbit or mouse secondary antibodies (1:300, Invitrogen, A-11008 anti-rabbit or A-11001 anti-mouse), and mounted with DAPI medium (Solarbio). The samples were observed under a fluorescence microscope (Olympus IX73), and the fluorescence intensity of the positively stained cells or tissue area was measured using Image J. 

### TUNEL assay

TUNEL staining was performed using the Apoptosis Detection Kit (Yeasen Biotech, Shanghai, China) [10]. Briefly, cells or tissue sections were fixed for 25 min and then permeabilized with proteinase K (20 μg/ml) for 20 min. After incubating with TdT for 1 h, the cells or tissue sections were counterstained with DAPI. The number and proportion of apoptotic cells were evaluated under a fluorescence microscope (Olympus IX73). 

### Statistical analysis

The SPSS 20. 0 software (SPSS, Chicago, IL, USA) was sed for statistical analyses. Data are presented as mean ± standard deviation (SD). One-way anlysis of variance was used to analyse multiple groups, and unpaired Student's *t*-test was used to analyse differences between two groups. 

*P* < 0. 05 was considered as statistically significant.

## Results

### MON enhanced the survival of IL-1β-treated chondrocytes

**Fig. 1:**
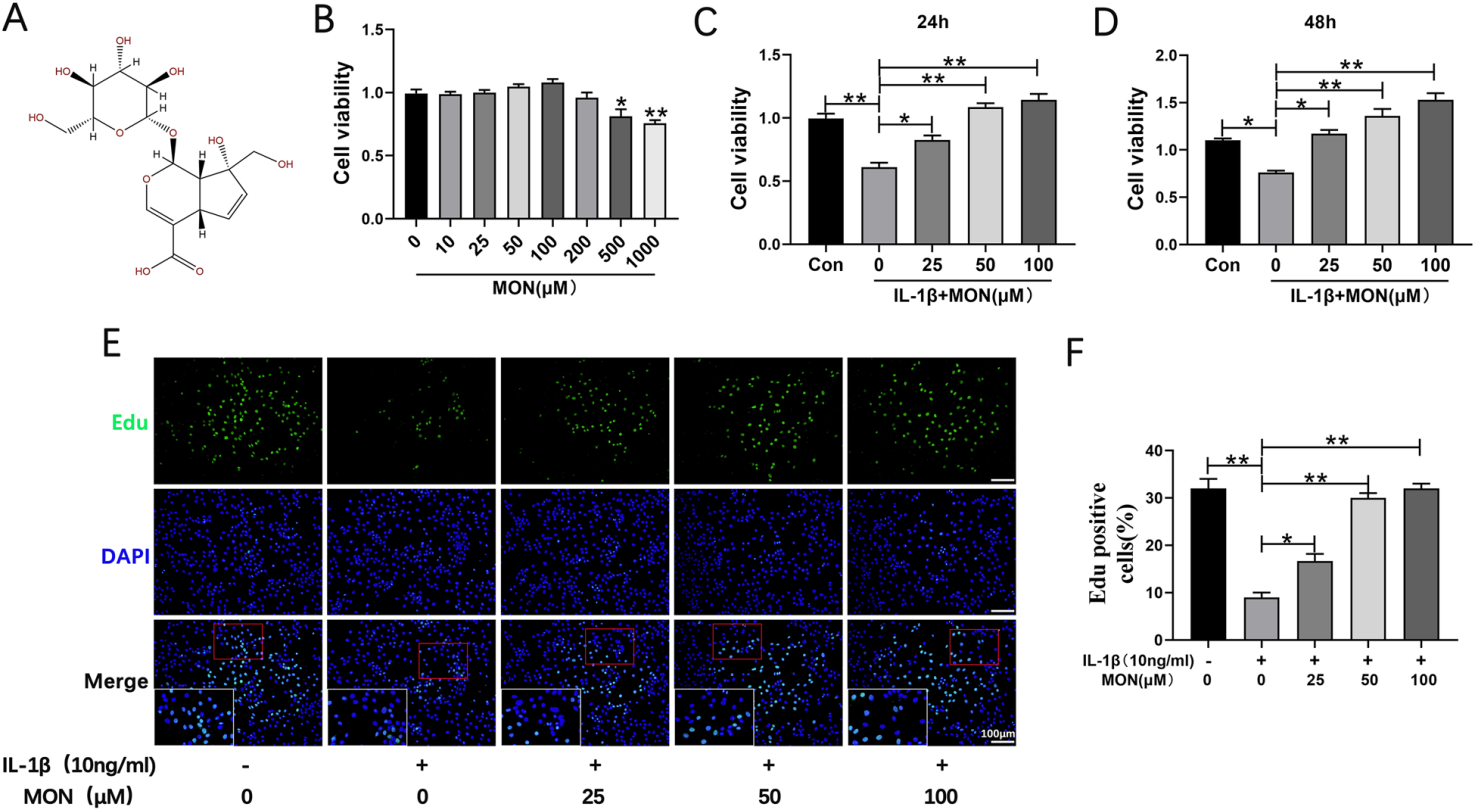
MON enhanced the survival of IL-1β-treated chondrocytes. **A** The molecular structure of MON. **B** The cell counting kit-8 assay showing the cell viability in different concentration of MON (0–1000 μM) for 24 h. **C**, **D** The cell counting kit-8 assay showing the cell viability of chondrocytes pretreatment with or without 10 ng/ml IL-1β for 24 h, followed by treatment with different concentration of MON (0, 25, 50 and 100 μM) for 24 h or 48 h. **E** Edu staining showing the proliferation of chondrocytes pretreated with or without 10 ng/ml IL-1β for 24 h, followed by treatment with different concentration of MON (0, 25, 50 and 100 μM) for another 24 h. **F** Quantification of Edu positive cells. All experiments were performed in triplicated and data were presented as the mean ± SD, *n* = 3 per group. *P < 0. 05, **P < 0. 01

To determine the effects of MON (chemical structure is shown in Fig. 1) on chondrocyte viability in vitro, primary mouse chondrocytes were treated with the different concentrations of MON with or without prior IL-1β stimulation. As shown in Fig. 1B, MON was non-toxic to the mouse chondrocytes at concentrations lower than 200 μM. Accordingly, 25, 50 and 100 μM doses were used in subsequent experiments. IL-1β stimulation markedly reduced the viability of chondrocytes, which was reversed by MON in a dose- and time-dependent manner (Fig. 1C, D). Consistent with this, Edu incorporation assay showed that MON significantly increased the proportion of the proliferating IL-1β-stimulated chondrocytes in a dose-dependent manner (Fig. 1E, F). In summary, MON treatment protected the chondrocytes against IL-1β, which is the major effector of the NLRP3 inflammasome.

### MON inhibited IL-1β-stimulated cartilage matrix degradation and apoptosis of chondrocytes

**Fig. 2:**
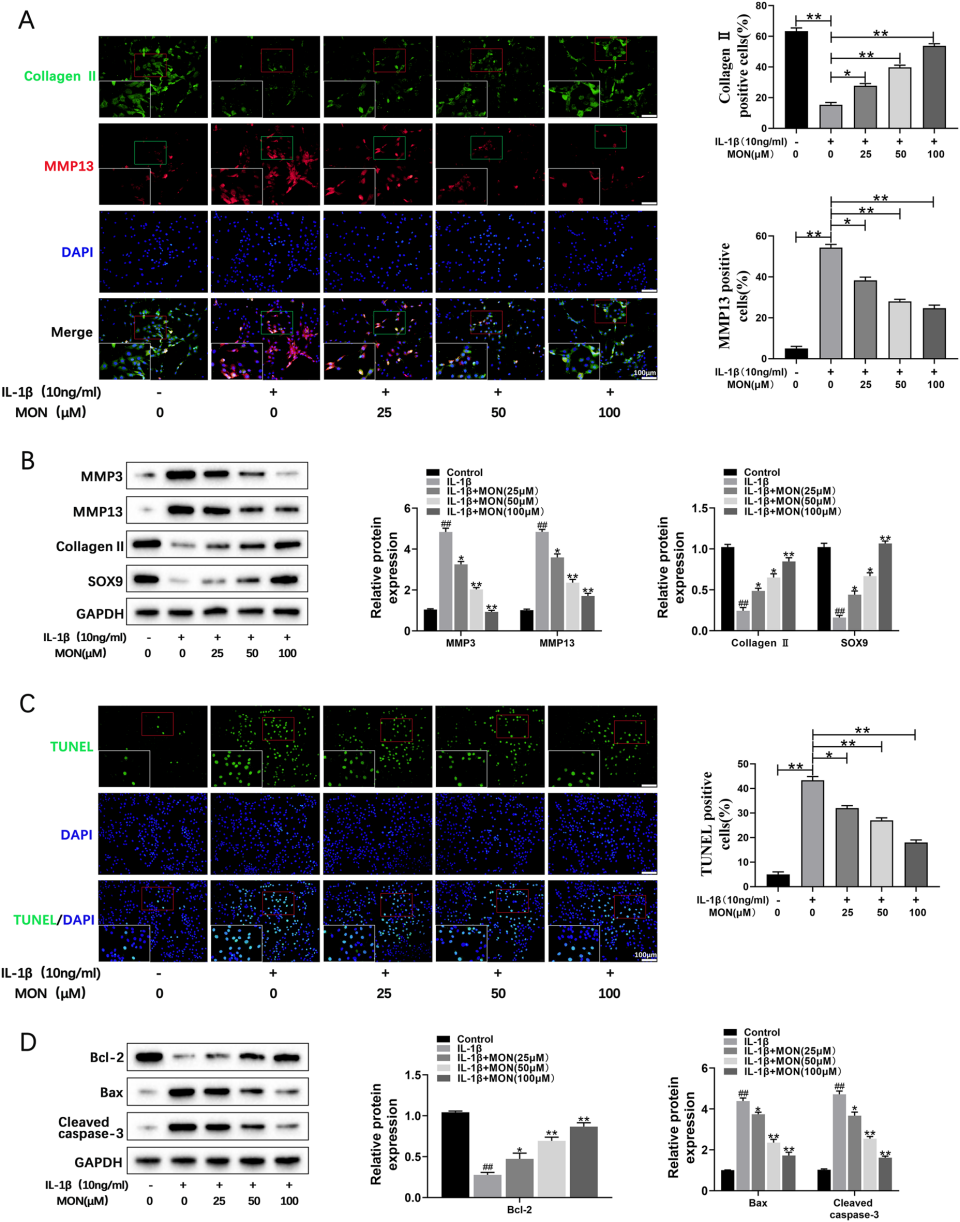
MON inhibited IL-1β-stimulated cartilage matrix degradation and apoptosis of chondrocytes. **A** Co-immunofluorescence staining of Collagen II (green)/MMP13 (red) and relative quantification of Collagen II or MMP13 positive cells in IL-1β-induced chondrocytes after MON treatment. **B** Western blot analysis and relative quantification showing the expression levels of cartilage matrix degradation related proteins (MMP3, MMP13, Collagen II and SOX9) in IL-1β-induced chondrocytes after MON treatment. **C** TUNEL staining showing the chondrocyte apoptosis and quantitative estimation of the number of TUNEL positive cells in IL-1β-induced chondrocytes after MON treatment. **D** Western blot analysis and relative quantification showing the expression levels of apoptosis related proteins (Bcl-2, Bax and Cleaved caspase-3) in IL-1β-induced chondrocytes after MON treatment. All experiments were performed in triplicated and data were presented as the mean ± SD, *n* = 3 per group. ^##^*P* < 0. 01 *versus* control group;*P < 0. 05, **P < 0. 01 *versus* IL-1β group

To further evaluate the protective effects of MON, we treated the IL-1β-stimulated primary chondrocytes with 0, 25, 50 and 100 μM MON for 24 h. As shown in Fig. 2A, IL-1β treatment decreased the in situ expression of collagen II and increased that of MMP13 in the primary chondrocytes. However, MON restored the levels of both matrix proteins in a dose-dependent manner. Analysis of the total protein fraction from the IL-1β-stimulated chondrocytes further showed that MON increased the levels of collagen II and SOX9, and decreased that of MMP3 and MMP13 (Fig. 2B). These findings suggested that MON can effectively reverse cartilage matrix degradation during chronic inflammation. TUNEL staining further showed that the number of apoptotic cells increased markedly after IL-1β treatment, which was mitigated by MON in a dose-dependent manner compared to the controls (Fig. 2C). Consistent with this, the anti-apoptotic Bcl-2 protein was significantly downregulated, whereas the pro-apoptotic Bax and cleaved caspase-3 proteins were upregulated following IL-1β exposure. MON treatment not only restored the expression levels of Bcl-2 but also downregulated Bax and cleaved caspase-3 (Fig. 2D). Taken together, MON inhibited IL-1β-stimulated degradation of the cartilage matrix and apoptosis of chondrocytes in vitro.

### MON inhibited IL-1β-induced pyroptosis in chondrocytes

**Fig. 3:**
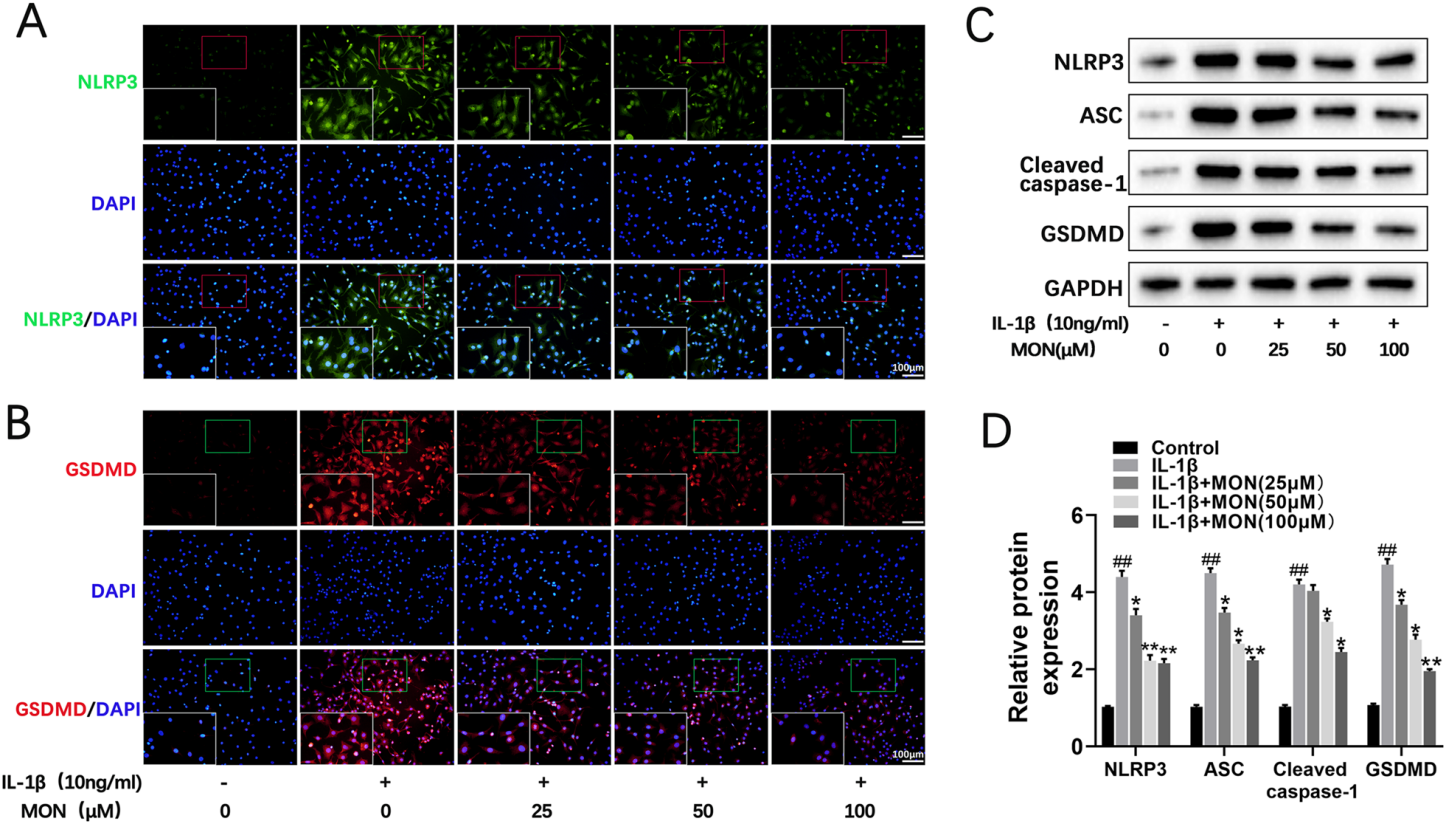
MON inhibited IL-1β-induced pyroptosis in chondrocytes. **A**, **B** Immunofluorescence staining of NLRP3 (green) and GSDMD (red) were performed respectively in IL-1β-induced chondrocytes after MON treatment. **C**, **D** Western blot analysis and relative quantification showing the expression levels of pyroptosis related proteins (NLRP3, ASC, Cleaved caspase-1 and GSDMD) in IL-1β-induced chondrocytes after MON treatment. All experiments were performed in triplicated and data were presented as the mean ± SD, *n* = 3 per group. ^##^*P* < 0. 01 *versus* control group;*P < 0. 05, **P < 0. 01 *versus* IL-1β group

Pyroptosis is a pro-inflammatory type of programmed cell death mediated by the gasdermin (GSDMD) protein [41, 42]. The binding of NLRP3 to the adapter protein ASC cleaves caspase-1, and activates IL-1β and IL-18. Furthermore, GSDMD cleavage results in the formation of membrane pores that release the inflammatory factors, eventually leading to pyroptosis [20, 30]. We found that IL-1β significantly increased the expression of NLRP3 and GSDMD in the primary chondrocytes, whereas MON treatment led to their downregulation in a dose-dependent manner (Fig. 3A, B). Western blotting further showed that IL-1β treatment markedly increased the expression of NLRP3, ASC, cleaved caspase-1 and GSDMD, which was reversed by MON in a dose-dependent manner (Fig. 3C, D). Collectively, these findings indicated that MON treatment can attenuate OA progression by inhibiting IL-1β-induced pyroptosis in chondrocytes.

### MON inhibited the NF-κB signaling pathway in chondrocytes

**Fig. 4:**
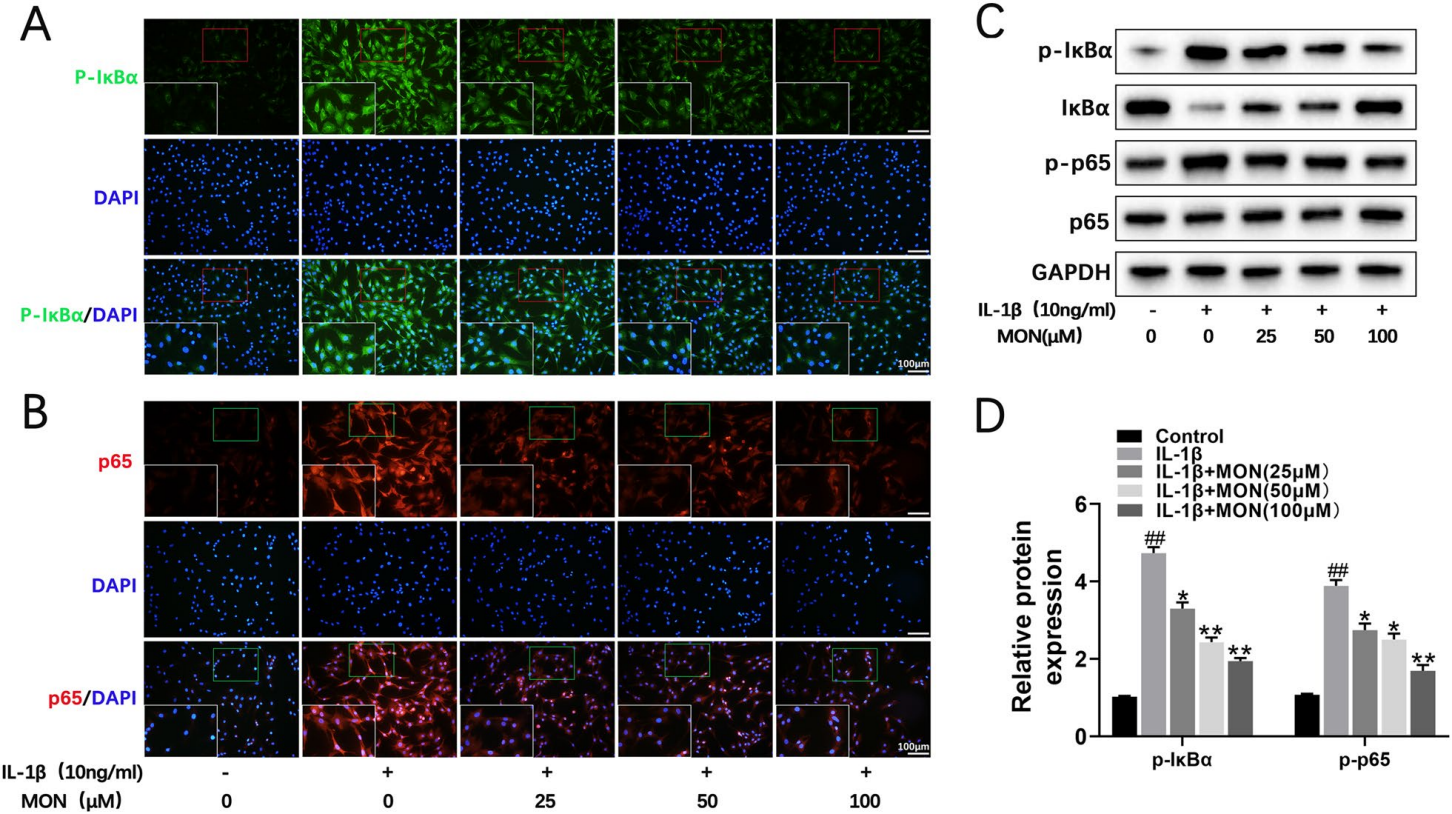
MON inhibited the NF-κB signaling pathway in chondrocytes. **A**, **B** Immunofluorescence staining showing the phosphorylation of IκBα (green) and the nuclear translocation of p65 (red) in IL-1β-induced chondrocytes after MON treatment. **C**, **D** Western blot analysis and relative quantification showing the expression levels of NF-κB signaling pathway related proteins (p-IκBα, IκBα, p-p65 and p65) in IL-1β-induced chondrocytes after MON treatment. All experiments were performed in triplicated and data were presented as the mean ± SD, *n* = 3 per group. ^##^*P* < 0. 01 *versus* control group;*P < 0. 05, **P < 0. 01 *versus* IL-1β group

Activation of the IKK complex phosphorylates the NF-κB inhibitor protein IκBα, which leads to its proteasomal degradation and the translocation of NF-κB dimers to the nucleus to drive transcription of target genes [21, 44, 46]. IL-1β treatment not only increased the expression of p-IκBα (Fig. 4A) but also promoted nuclear translocation of p65 (Fig. 4B) in the primary chondrocytes, whereas MON reversed these effects of IL-1β in a dose-dependent manner (Fig. 4A, B). Furthermore, IL-1β also upregulated p-IκBα and p-p65 and activated the NF-κB pathway, while MON decreased the phosphorylation of IκBα and p65 without affecting the total p65 levels (Fig. 4C, D). Taken together, MON can inactivate the NF-κB pathway in IL-1β-stimulated chondrocytes.

### The protective effects of MON are mediated through the inactivation of NF-κB signaling

**Fig. 5:**
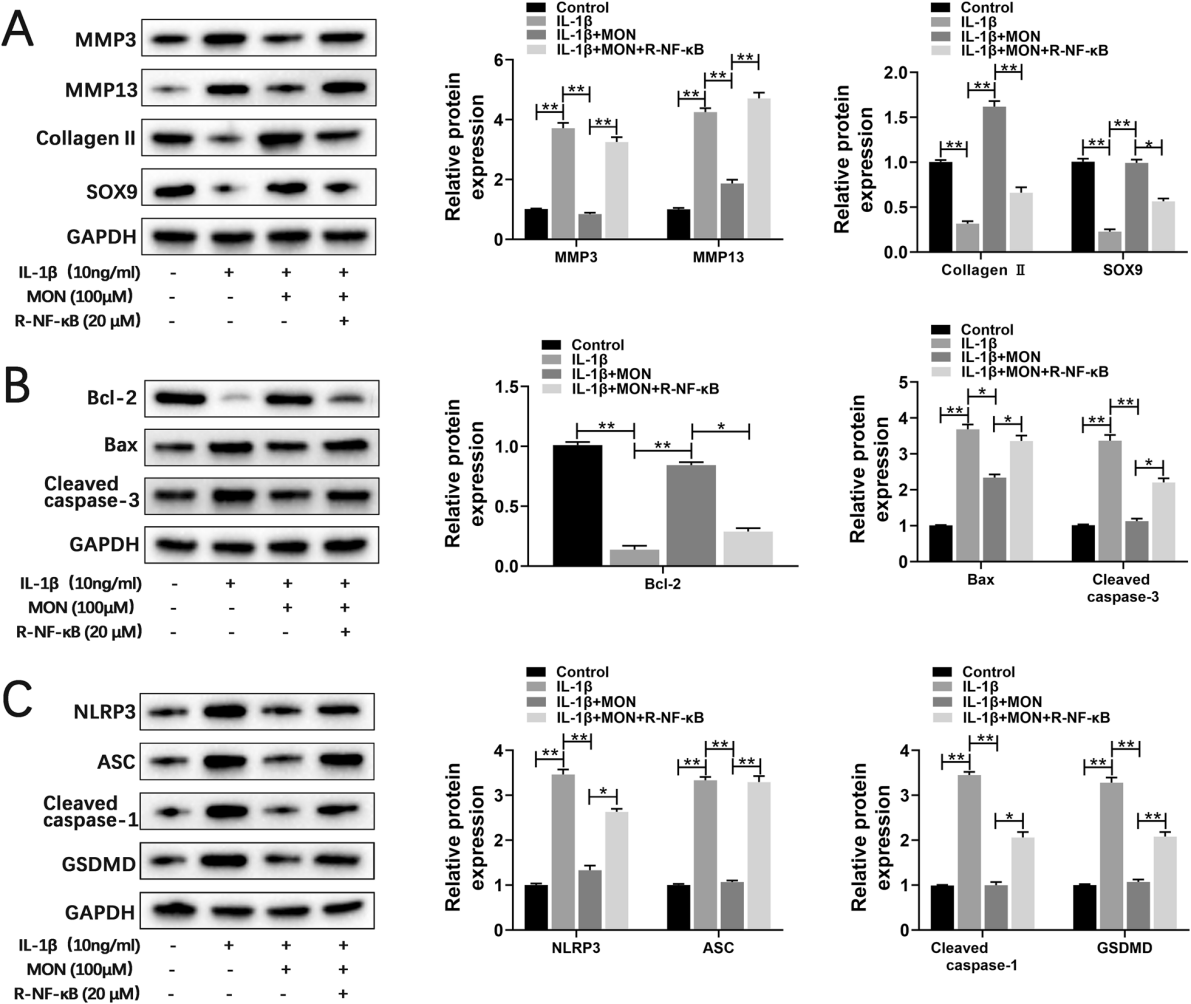
The protective effects of MON are mediated through the inactivation of NF-κB signaling. **A**–**C** Western blot analysis and relative quantification showing the expression levels of cartilage matrix degradation related proteins (MMP3, MMP13, Collagen II and SOX9), apoptosis related proteins (Bcl-2, Bax and Cleaved caspase-3) and pyroptosis related proteins (NLRP3, ASC, Cleaved caspase-1 and GSDMD) in IL-1β-induced chondrocytes after MON and recombinant NF-κB protein treatment. All experiments were performed in triplicated and data were presented as the mean ± SD, *n* = 3 per group. *P < 0. 05, **P < 0. 01

To next determine whether the NF-κB pathway is critical to the effects of MON on the cartilage matrix proteins and chondrocytes, we stimulated the primary chondrocytes with 10 ng/ml IL-1β and 20 μM recombinant NF-κB (R-NF-κB, Cloud-Clone Corp) before treating them with 100 μM MON. Recombinant NF-κB reversed the effects of MON on MMP3, MMP13, collagen II and SOX9 (Fig. 5A), as well as on the expression levels of apoptosis markers (Fig. 5B) in the IL-1β-stimulated cells. Furthermore, recombinant NF-κB also enhanced the expression of pyroptosis proteins including NLRP3, ASC, cleaved caspase-1 and GSDMD despite MON treatment (Fig. 5C). Collectively, these findings demonstrated that MON inhibited IL-1β-induced cartilage matrix degradation, and the apoptosis and pyroptosis of chondrocytes by targeting the NF-κB pathway.

### MON alleviated arthritic progression and promoted cartilage repair in the DMM model by targeting the NF-κB pathway

**Fig. 6:**
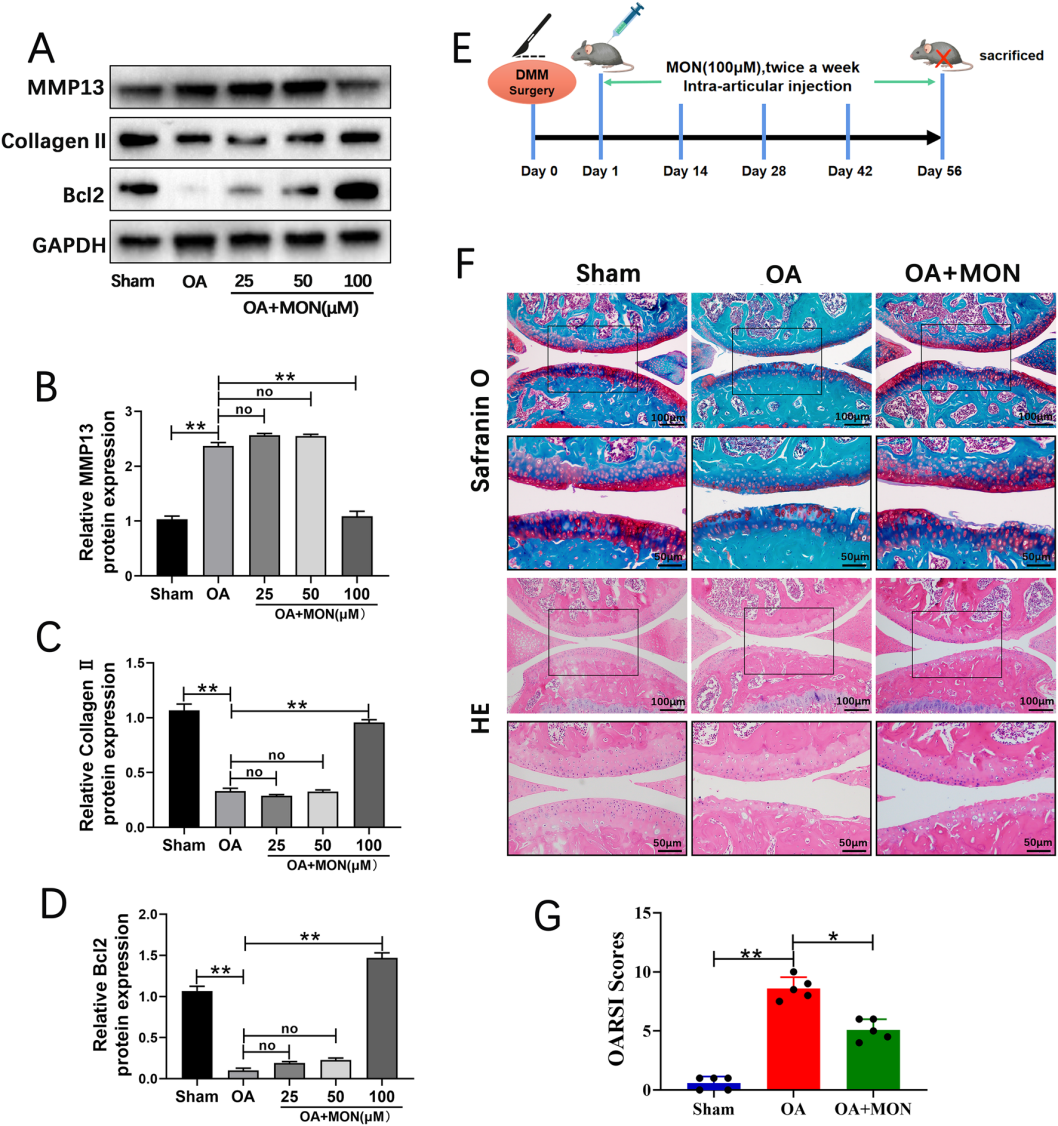
MON alleviated OA progression and promoted cartilage repair in DMM mice. **A**–**D** The pre-experimental results suggested that the concentration of 100 μM MON showed significant statistical difference. **E** DMM mice model and MON treatment protocol. **F** Safranin O-Fast Green and HE staining of mice knee joints were performed to assess the histological morphology of the different groups at 56 days after MON treatment. **G** The OARSI scores of the different groups were calculated respectively at 56 days after MON treatment. All experiments were performed in triplicated and data were presented as the mean ± SD, *n* = 3 per group. *P < 0. 05, **P < 0. 01

**Fig. 7:**
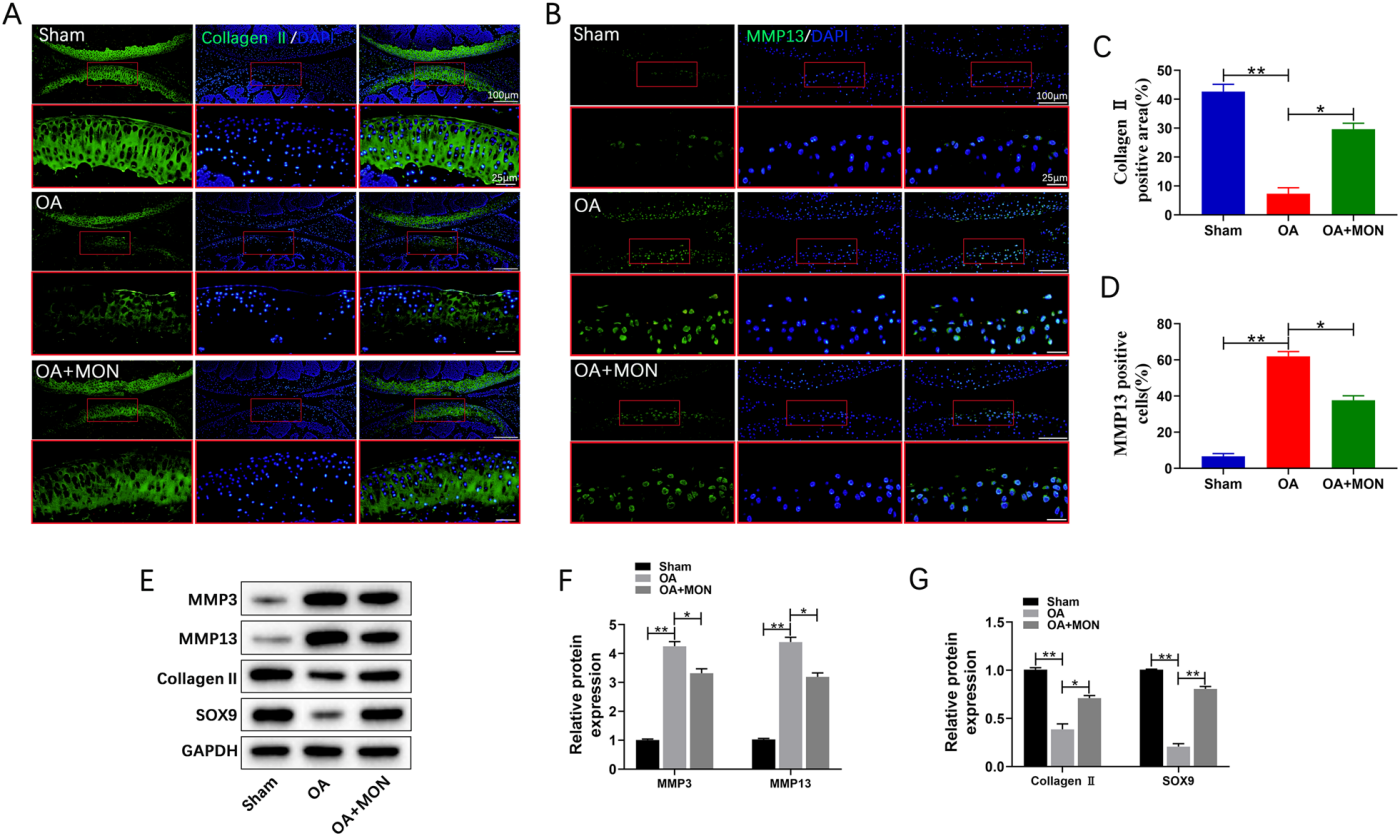
MON inhibited cartilage matrix degradation in DMM mice. **A**, **B** Immunofluorescence staining of Collagen II and MMP13 were performed respectively in mice knee joints at 56 days after MON treatment. **C**, **D** The relative quantification analysis of Collagen II positive area and MMP13 positive cells. **E**–**G** Western blot analysis and relative quantification showing the expression levels of cartilage matrix degradation related proteins (MMP3, MMP13, Collagen II and SOX9) in DMM mice at 56 days after MON treatment. All experiments were performed in triplicated and data were presented as the mean ± SD, *n* = 3 per group. *P < 0. 05, **P < 0. 01

**Fig. 8:**
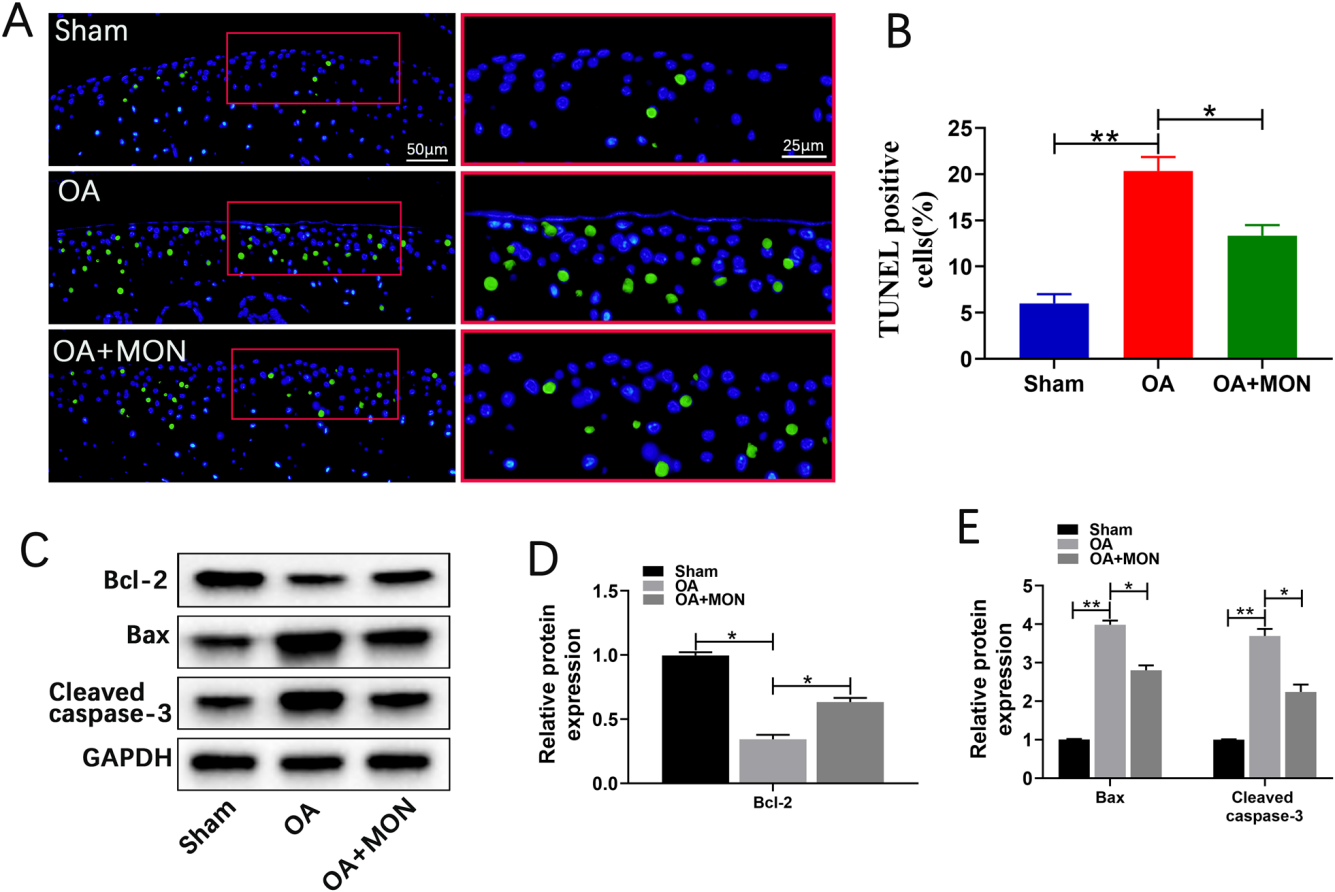
MON inhibited chondrocyte apoptosis in DMM mice. **A**, **B** TUNEL staining showing the chondrocyte apoptosis and quantitative estimation of the number of TUNEL positive cells in mice knee joints at 56 days after MON treatment. **C**–**E** Western blot analysis and relative quantification showing the expression levels of apoptosis related proteins (Bcl-2, Bax and Cleaved caspase-3) in DMM mice at 56 days after MON treatment. All experiments were performed in triplicated and data were presented as the mean ± SD, *n* = 3 per group. *P < 0. 05, **P < 0. 01

**Fig. 9:**
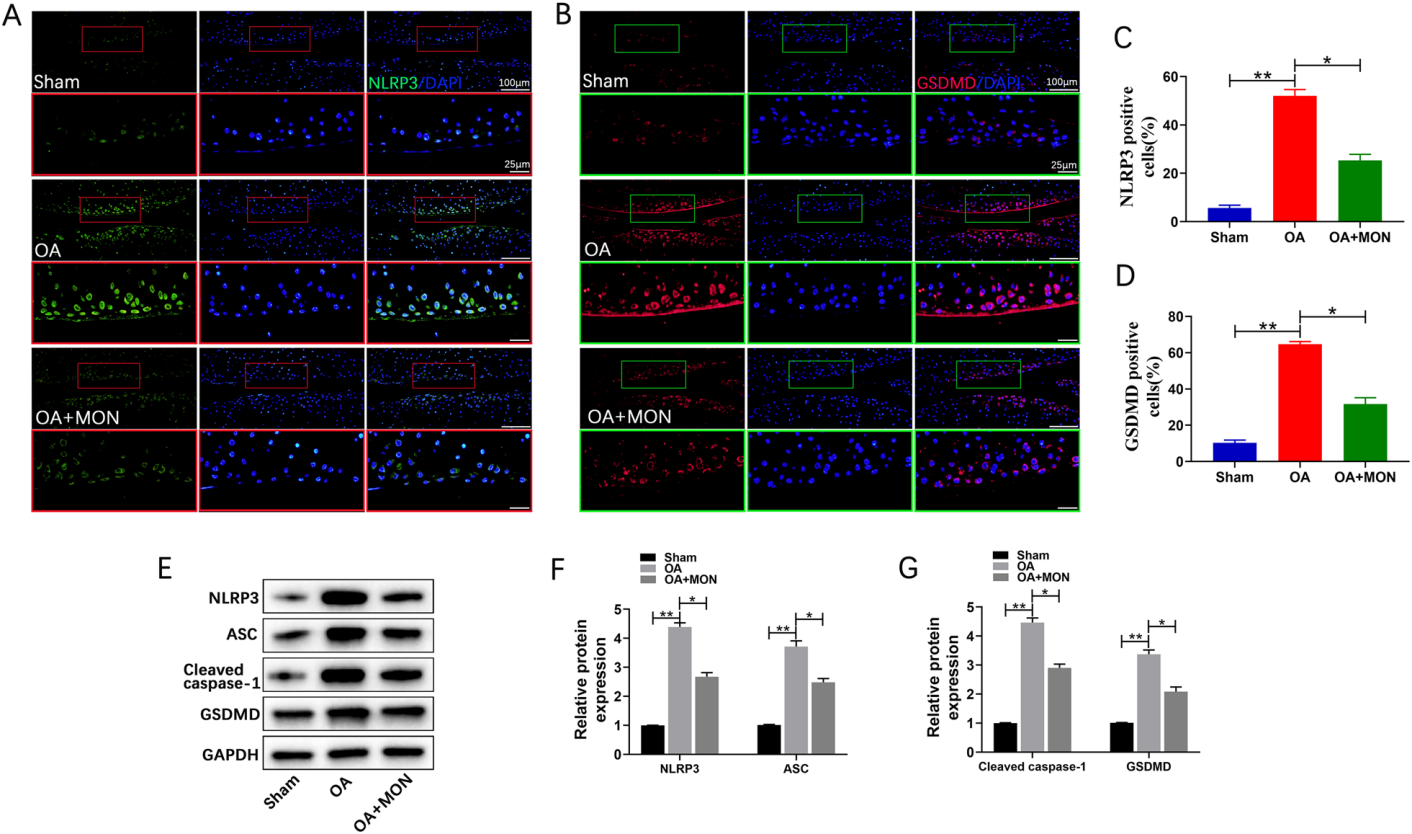
MON inhibited chondrocyte pyroptosis in DMM mice. **A**, **B** Immunofluorescence staining of NLRP3 (green) and GSDMD (red) were performed respectively in mice knee joints at 56 days after MON treatment. **C**, **D** The relative quantification analysis of NLRP3 and GSDMD positive cells. **E**–**G** Western blot analysis and relative quantification showing the expression levels of pyroptosis related proteins (NLRP3, ASC, Cleaved caspase-1 and GSDMD) in DMM mice at 56 days after MON treatment. All experiments were performed in triplicated and data were presented as the mean ± SD, *n* = 3 per group. *P < 0. 05, **P < 0. 01

The pre-experimental results suggested that the concentration of 100 μM MON showed significant statistical difference, the single-dose concentration was used in subsequent animal experiments (Fig. 6A–D). To determine the effects of MON on OA progression in vivo, the mice were injected with 100 μM MON twice a week into the operated knee for 8 weeks after OA induction (Fig. 6E). Safranin O-Fast Green and HE staining of the knee joint sections of arthritic mice indicated that DMM eroded the articular cartilage, which was attenuated by MON treatment (Fig. 6F). Furthermore, the OARSI scores also decreased significantly in the MON-treated mice compared to the untreated controls, which confirmed that MON reversed the cartilage damage associated with OA (Fig. 6G). Consistent with the gross findings, collagen II deposition decreased significantly and the number of MMP13-positive cells increased in the OA mice. However, MON treatment increased collagen II levels and decreased that of MMP13 (Fig. 7A–D). Analysis of the total protein levels of the cartilage tissues also indicated that MON treatment reversed the increase in MMP3 and MMP13, and the decrease in collagen II and SOX9 in the arthritic mice (Fig. 7E–G), which was consistent with the in vitro results. TUNEL staining of the cartilage sections further showed that a significant increase in the proportion of apoptotic chondrocytes was observed in the OA group, which was accompanied by elevated Bax and cleaved caspase-3 levels, and the downregulation of Bcl2. Intra-articular injection of MON alleviated chondrocyte apoptosis and restored the levels of the related markers (Fig. 8A–E). In line with the in vitro results, the pyroptosis-related markers including NLRP3, ASC, cleaved caspase-1 and GSDMD were elevated significantly in the OA mice compared to the sham-operated controls, and were reversed by MON treatment (Fig. 9A–G). Taken together, MON treatment alleviated the structural damage to the cartilage due to DMM and promoted chondrocyte survival, thereby halting arthritic progression.

**Fig. 10:**
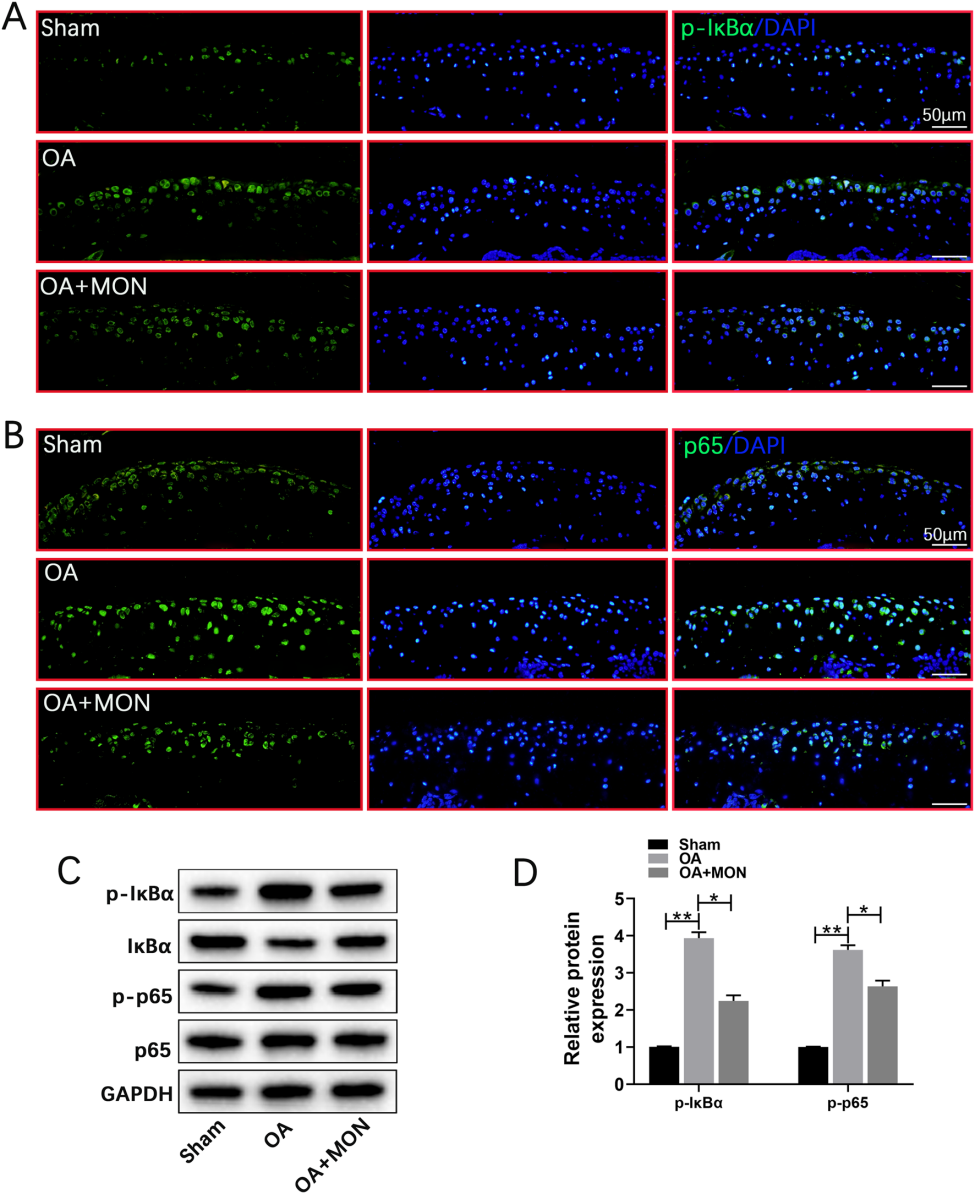
MON inhibited the activation of NF-κB signaling pathway in DMM mice. **A**, **B** Immunofluorescence staining showing the phosphorylation of IκBα (green) and the nuclear translocation of p65 (green) in mice knee joints at 56 days after MON treatment. **C**, **D** Western blot analysis and relative quantification showing the expression levels of NF-κB signaling pathway related proteins (p-IκBα, IκBα, p-p65 and p65) in DMM mice at 56 days after MON treatment. All experiments were performed in triplicated and data were presented as the mean ± SD, *n* = 3 per group. *P < 0. 05, **P < 0. 01

**Fig. 11:**
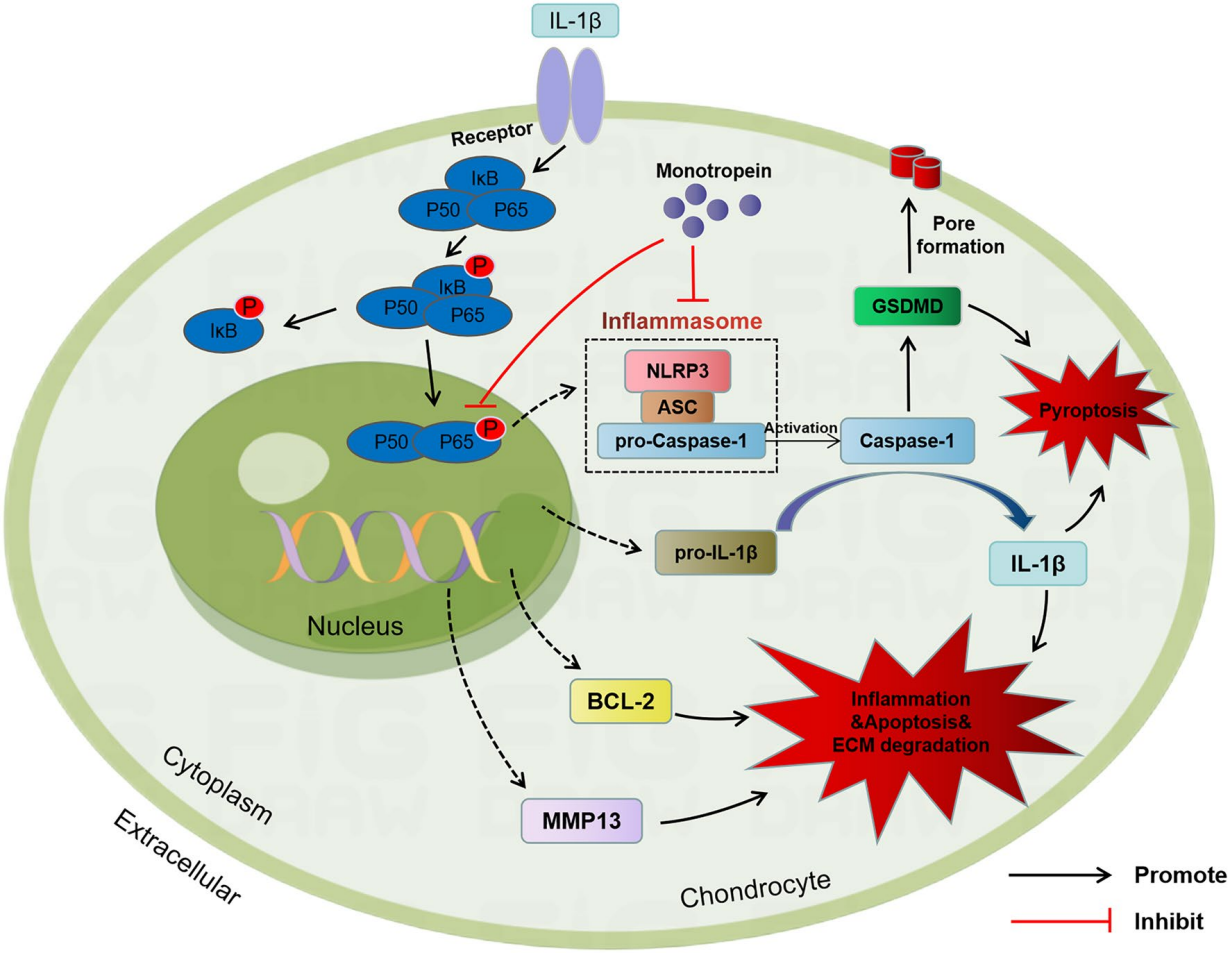
A schematic diagram illustrating the theory in the current study. MON inhibited cartilage matrix degradation and alleviated apoptosis and pyroptosis in the in vitro and in vivo models of OA by targeting the NF-κB signaling pathway

To ascertain the molecular basis of the effects of MON in vivo, we analyzed IκBα and p65 activation in the articular cartilage of the arthritic mice. As expected, DMM significantly increased the levels of p-IκBα and p-p65, as well as the nuclear translocation of p65 compared to the sham-operated group, which was reversed by MON treatment (Fig. 10A, B). Furthermore, MON treatment decreased the expression of p-IκBα and p-p65 without affecting total p65 levels in the knee joints of arthritic mice (Fig. 10C, D). In summary, MON protected against cartilage erosion after DMM by targeting the NF-κB signaling pathway. Moreover, A schematic diagram illustrating the theory in the current study can be found in Fig. 11.

## Discussion

Osteoarthritis (OA) is a chronic inflammatory disease of the joints that is characterized by the destruction of articular cartilage [37]. Extracellular matrix degradation, chondrocyte apoptosis and pyroptosis form the pathological basis of OA progression. The current treatment strategies for OA can only provide transient relief in the symptoms but cannot effectively prevent disease occurrence or progression [18, 28]. Hence, it is essential to develop therapeutic strategies that can enhance cartilage repair and block OA progression. 

MON, a bioactive compound of the medicinal herb *Morinda officinalis*, has documented anti-inflammatory, anti-oxidant, anti-apoptotic and autophagic effects. MON alleviated inflammation, oxidative stress and apoptosis associated with acute kidney injury by inhibiting NF-κB signaling [45]. In addition, MON attenuated liver injury in a mouse model via inactivation of the NF-κB pathway and NLRP3 inflammasome [4], and also decreased H_2_O_2_-induced oxidative stress by enhancing autophagy in osteoblasts [31]. Furthermore, previous studies have proved that MON could exert protective effects against IL-1β-induced apoptosis and catabolic responses in chondrocytes [34]. In the present study, we found that MON inhibited cartilage matrix degradation and promoted the survival of chondrocytes in the in vitro and in vivo models of OA by targeting the NF-κB signaling pathway. 

Excessive degradation of articular cartilage plays a crucial part in the pathogenesis of OA [25, 48]. The imbalance between extracellular matrix synthesis and degradation is the direct cause of articular cartilage erosion [8, 29]. OA is characterized by progressive destruction of cartilage, culminating in complete loss of chondrocytes. Moreover, collagen II and MMPs are thought to be the major contributors to cartilage degeneration during OA pathogenesis [25, 26]. Therefore, inhibiting cartilage matrix degradation and restoring its synthesis may ameliorate OA progression. We found that MON treatment upregulated the matrix proteins collagen II and SOX9, and down-regulated MMP3 and MMP13 in the IL-1β-stimulated chondrocytes as well as in the articular cartilage of OA mice. Thus, MON can mitigate OA progression by inhibiting cartilage matrix degradation. These findings are in accordance with previous studies indicating that MON upregulated COL2A1 and downregulated MMPs in the chondrocytes [34]. 

The loss of chondrocytes in the arthritic joints is attributed to apoptosis, which also results in the destruction of the cartilage homeostasis and the degeneration of articular cartilage [13, 32]. Therefore, promoting chondrocyte survival and inhibiting apoptosis can inhibit cartilage degeneration. MON has been previously demonstrated to attenuate apoptosis in IL-1β-stimulated chondrocytes [34]. Similar results were obtained in our study as well, and the anti-apoptotic effects of MON were accompanied by an increase in the expression of Bcl-2, and decrease in Bax and cleaved caspase-3 levels in the IL-1β-induced chondrocytes and mouse model. Thus, MON alleviated OA progression by inhibiting chondrocyte apoptosis. This is consistent with a previous finding that MON inhibited apoptosis in senescent endothelial cells and an acute kidney injury mice model by targeting NF-κB signaling [15, 45]. 

Pyroptosis, a pro-inflammatory type of programmed cell death mediated by the NLRP3 inflammasome, has been implicated in the pathological progression of OA [24, 39]. Activation of NLRP3 cleaves the caspase-1 precursor to accelerate the release of inflammatory factors into the extracellular space [1]. Pro-inflammatory factors produced during pyroptosis of chondrocytes or synovial macrophages can directly lead to synovial inflammation and the degradation of cartilage matrix, and thus promote OA progression [1, 43]. MON treatment dramatically down-regulated the pyroptosis-related proteins in IL-1β-stimulated chondrocytes and DMM-induced mice, suggesting that inhibition of chondrocyte pyroptosis may be a potential mechanism by which MON alleviates OA progression. Consistent with these results, MON attenuated liver injury by down-regulating the activity of NLRP3 inflammasome [4]. Furthermore, Zhang et al. also reported that the NLRP3 inhibitor CY-09 protected chondrocytes against inflammation and alleviated OA progression by blocking NLRP3 inflammasome-mediated pyroptosis [44, 46]. Another study showed that Icariin alleviated LPS-induced chondrocyte pyroptosis by inhibiting NLRP3/caspase-1 signaling pathway, and mitigated the symptoms of OA [49]. Therefore, the NLRP3 inflammasome is a promising therapeutic target for OA. 

Apoptosis is a process of programmed cell death that does not cause any inflammatory response, in contrast, pyroptosis is the programmed inflammatory cell death mediated by the NLRP3 inflammasome and releases plenty of pro-inflammatory factors [1, 13, 39]. It has been proved that apoptosis and pyroptosis of chondrocytes mediate inflammation of cartilage or synovium, aggravate cartilage matrix degradation, break the dynamic balance of extracellular matrix synthesis and degradation, and aggravate the development of OA [13, 39]. Thus, chondrocyte apoptosis and pyroptosis as well as cartilage matrix degradation are crucial pathological changes in OA. 

Numerous previous studies have demonstrated that the NF-κB pathway is a classical inflammatory pathway involved in OA development [5]. Activation of the NF-κB pathway culminates in the production of pro-inflammatory cytokines such as IL-1β, TNFα, etc. , which induces chondrocyte apoptosis and pyroptosis, promotes cartilage matrix degradation, and aggravates OA progression [16]. Therefore, inhibiting NF-κB pathway activation and reducing inflammatory response can aid in protecting cartilage tissues and alleviating OA progression. In our study as well, the NF-κB pathway was activated in both IL-1β-stimulated chondrocytes and DMM-induced mice, and MON treatment blocked the pathway via inhibiting the phosphorylation of IκBα and nuclear translocation of p65. Furthermore, the recombinant NF-κB reversed the therapeutic effects of MON, indicating that the NF-κB signaling pathway is critical to the role of MON in the chondrocytes. Previous studies have also shown that MON inactivated the NF-κB pathway in different disease models, such as chronic colitis and acute kidney injury [4, 45]. Jiang et al. [15] reported that MON alleviated inflammation, oxidative stress and apoptosis in senescent endothelial cells by inhibiting NF-κB signaling. 

The regulatory relationship between NF-κB signaling pathway and pyroptosis has been elucidated in recent years [22, 41, 42]. NF-κB lies upstream of NLRP3 and induces its transcription [7, 22, 35]. Activation of the NF-κB pathway dissociates NF-κB from IκB, and the free NF-κB translocates to the nucleus and transcriptionally activates the NLRP3 inflammasome, thus promoting the release of IL-1β and IL-18, which leads to chondrocyte pyroptosis [14, 38, 40]. Previous studies have shown that Moroniside alleviated chondrocyte pyroptosis in OA mice by reducing the levels of NLRP3 inflammasome and caspase-1 via NF-κB inhibition [41, 42]. Another study showed that Loganin significantly inhibited the NF-κB pathway and decreased the levels of cryopyrin and caspase-1 in OA mice, which prevented chondrocyte pyroptosis [11]. Therefore, targeted inhibition of the NF-κB signaling pathway may protect chondrocytes from pyroptosis and thus alleviate OA progression. 

## Limitations

However, our current study also has some limitations. First of all, we used positive control drug in the preliminary experiment, however, there were no positive drug for OA mouse model in the formal experiment. Furthermore, micro-CT were not used for the evaluation of the bone situation especially cartilage erosion due to lack of funding. Further studies will be performed by us in the future to refine these limitations. 

## Conclusion

In summary, MON inhibited cartilage matrix degradation and alleviated apoptosis and pyroptosis in the in vitro and in vivo models of OA by targeting the NF-κB signaling pathway. Therefore, MON is a promising drug for promoting cartilage repair and stalling OA progression, and warrants clinical validation. 

## Data Availability

The data used to support the findings of this study are available from the corresponding author upon request.

## Abbreviations

ASC: Apoptosis-associated speck-like protein containing a CARD
BCA: Bicinchoninic acid
CCK-8: Cell counting kit-8
DMEM: Dulbecco’s modified eagle medium
DMM: Destabilization of the medial meniscus
EDTA: Ethylenediaminetetraacetic acid
EdU: Ethynyl-deoxyuridine
FBS: Fetal bovine serum
GSDMD: Gasdermin D
HE: Hematoxylin–eosin
MMP13: Matrix metallopeptidase 13
MMP3: Matrix metallopeptidase 3
MON: Monotropein
NLRP3: NLR family pyrin domain containing 3
OA: Osteoarthritis
OARSI: Osteoarthritis Research Society International
PBS: Phosphate-buffered saline
p-IκBα: Phosphorylated IκBα
PVDF: Polyvinylidene difluoride
RIPA: Radio Immunoprecipitation Assay
R-NF-κB: Recombinant NF-κB
RT: Room temperature
SDs: Standard deviations
SPF: Specific pathogen-free
TBST: Tris buffer solution
TLR_4_: Toll-like receptor 4
